# HPV-Associated Breast Cancer: Myth or Fact?

**DOI:** 10.3390/pathogens11121510

**Published:** 2022-12-09

**Authors:** Erik Kudela, Eva Kudelova, Erik Kozubík, Tomas Rokos, Terezia Pribulova, Veronika Holubekova, Kamil Biringer

**Affiliations:** 1 Clinic of Gynecology and Obstetrics, Jessenius Faculty of Medicine, Comenius University in Bratislava, 03601 Martin, Slovakia; 2 Clinic of Surgery and Transplant Center, Jessenius Faculty of Medicine, Comenius University in Bratislava, 03601 Martin, Slovakia; 3 Biomedical Center Martin, Jessenius Faculty of Medicine, Comenius University in Bratislava, 03601 Martin, Slovakia

**Keywords:** human papillomavirus, breast cancer, oncogene, carcinogenesis

## Abstract

Some estimates place the proportion of human malignancies attributable to viruses at between 15 and 20 percent. Viruses including the human papillomavirus are considered an interesting but controversial etiological risk factor for breast cancer. HPV infection is anticipated to be an early trigger in breast cancer carcinogenesis, followed by cumulative alterations over time (“hit and run” mechanism) through synergy with other environmental factors. The association between HPV and breast cancer has not yet been verified. There are very conflicting data on the presence of HPV DNA in breast cancer samples, and we lack a clarified, exact mode of HPV transmission to the breast. In our review article we analyzed the up-to-date knowledge about the association of HPV and breast cancer. Furthermore, we summarized the available original research published since 2010. In conclusion, the complexity and inconsistency of the available results together with the relatively low prevalence of HPV infection requires extensive research with much larger studies and exact and unified diagnostic methods are required to better understand the role of the HPV in breast carcinogenesis.

## 1. Introduction

The number of newly diagnosed cases of breast cancer (BC) has been predicted at 2.3 million in 2020 with mortality close to 685,000. BC incidence and mortality are predicted to rise to 4.4 million and 2.1 million, respectively, by 2070 [1,2]. BC is now the world’s most frequently diagnosed cancer and the fifth leading cause of cancer mortality. In most countries, BC accounted for approximately 24.5% of all cancer diagnoses among women and 15.5 % of all cancer deaths in 2020 (15.5%) [1]. Although the average age of breast cancer patients is 61 years, approximately one in forty women is diagnosed with the disease at a young age (≤40 years old) [3,4].The development of breast cancer can be attributed to several factors, including advanced age, high body mass index or obesity, tobacco use, physical inactivity, a high-fat diet, early menarche, late age at first full-term pregnancy, shorter breastfeeding periods, the use of hormonal replacement therapy or oral contraceptives, breast density, and family history of breast cancer [5]. Despite the tremendous effort that has been made to explore the etiology of breast cancer, the identified risk factors explain only a portion of malignant tumors.

Viruses are considered an interesting but controversial etiological risk factor for breast cancer, which can precipitate tumorigenesis via synergy with other environmental factors. There is evidence that viral DNA from human papillomaviruses (HPV), Epstein–Barr virus (EBV), human cytomegalovirus (HCMV), herpes simplex virus (HSV), and human herpesvirus type 8 (HHV-8) can be found in breast cancer samples as well as in healthy tissue samples [6,7]. However, there is no confirmation of viral breast carcinogenesis; the available data show no trend, even within the same nation, and some of them are contradictory [8].

In our review article, we focused on the oncogenic potential of HPV and its link to breast cancer. We searched the PubMed database using the keywords “breast cancer”, “HPV”, and “human papillomavirus”. In the literature search, all literature up to October 2022 was included, with no restriction on the publication date.

## 2. Mechanisms of HPV Infection Targeting Breast Tissue

Two main hypotheses have been proposed about how HPV can infect mammary gland cells; however, the specific mechanism is still unknown. According to the first hypothesis, HPV is transmitted to the mammary glands via the lymphatic or blood system through mononuclear white cells present in women with cervical dysplasias. In the case of malignancy, cells from a primary tumor can be transported by plasma flow. In addition, HPV virions can be transferred from the site of initial infection to other organs [9]. Other scientists argue that HPV viremia is improbable, since the HPV lifecycle occurs in the epithelial layers [10].

De Villiers et al. [11], who postulated a retrograde ductal pattern of viral propagation, provided evidence in support of the second theory, which states that HPV may infect the mammary gland through the skin of the nipple. Since the milk ducts are open ducts, they might serve as an entrance route for viral infection, and so the risk of HPV infection is higher when they are exposed to the external environment. The most common way of transmission for HPV is sexual contact, with the virus potentially entering via nipple fissures or by hand-mediated contact between the female perineum and the breast during sexual intercourse [7,12]. It should be noted that most HPV-positive children, infected via the placenta or during vaginal delivery [13,14], have the same type of HPV that is in the mother’s genital region at birth. If newborns may be involved in the virus’s transfer to the breast region [15,16] remains controversial because of the presence of HPVs also in breast milk of HPV infected mothers [17,18].

### Virus Entry to the Breast Cells

One hypothesis is based on the complex mechanism of endocytosis and cellular transport associated with alpha HPV genus (annexins, integrins, tetraspanins and EGFRs) [19]. The α6 integrins are recognized as the main receptors for HPV 16 in uterine cervix cells [20] and, in the case of breast tissue together with laminin-322, they are essential for normal mammary morphogenesis. The results of particular studies suggest that they can act as HPV receptors during infection and promote tumor progression during infection [21,22]. The signals from integrin receptors are further essential for tumor cell growth, apoptosis, angiogenesis and metastatic process [23]. After the cell entrance, the viral genome can be found in episomal form [24,25]. Whether the cell entry mechanism in low-risk HPVs (lrHPVs) is different remains questionable.

Another hypothesis is based on the activity of the extracellular vesicles (EVs). The ascending pattern of HPV infection spread is still a target of much discussion. The presence of HPV DNA in serum-derived EVs was found in patients with HPV DNA-positive squamous cell carcinoma of the middle rectum and BC patients [26]. EVs include exosomes, microvesicles and apoptotic bodies, which differ in size and biophysical characteristics [27].

Many types of cells, tissues and bodily fluids produce EVs, including nucleic acids, proteins, non-coding RNA and viral nucleic acids [28]. Furthermore, several authors have observed that oxidative stress and radiation-induced DNA damage may impact stromal compartment and EV absorption [29]. The content of HPV-positive EVs produced from a primary site of infection may be transferred to cells without HPV receptors (e.g., fibroblasts and breast epithelial cells) and cause a local induction of tumor cell proliferation. In situ hybridization revealed that HPV DNA was present both in the epithelium and in the stroma [30].

## 3. Molecular Base of HPV-Associated Breast Cancer Carcinogenesis

The genome of HPV consists of double-stranded circular DNA reaching the length of around 8000 base pairs, containing from eight to nine open-reading fragments (ORFs). It consists of three functional regions: a long control region (LCR), early region (E ORF) and late region (L ORF). The approximately 1 kb long section of the LCR has no coding potential and contains sections regulating viral transcription and tissue tropism. Early region is represented by 6–7 ORFs (E1, E2, E4, E5, E6, E7 and E8), whereas the late region consists of only two ORFs (L1 and L2). All HPVs contain conserved core genes included in replication (E1 and E2) and viral capsid establishment (L1 and L2), with more diversity in other genes (E4, E5, E6 and E7), which determine maturation and release of the virus, escape from immune system and regulation of cell cycle [31].

The majority of HPV genomes (86–100%) detected in breast tissue are in an integrated form with a low copy number (0.00054–9.3 copies/cell) [12]. In BC, HPV is suspected to play only an indirect role in these malignancies due to the low viral burden. Multiple studies around the world have investigated an exotic relationship between the HPV and breast cancer, but their detection of HPV DNA (e.g., L1, L2, E1, E2, E6 and E7) in mammary tumors has been extremely variable (from 0 to 86.2%) and does not depend on the women’s age [32,33] (Table 1 and Table 2).

Molecular changes in BC initiation may therefore occur via a “hit and run” mechanism. This theory proposes that HPV initiates or contributes to the development of cancer, but in some cases vanishes from tumor cells (possibly due to immune surveillance) before the disease is diagnosed [45,61]. The possible role of HPV as a mediator or cofactor in a causal relationship remains to be determined by future research.

As a result of the integration of the HPV genome into the host genome, chromosomal instability and carcinogenesis may be induced [92]. HPV E6 and E7 oncogenes were identified in BC samples, suggesting that HPV may be involved in the promotion of BC. The E6/E7 mRNA was found in 24–100% of HPV-positive BC samples. Moreover, new fusion transcripts of E6/E7 (E6^E7*I, E6^E7*II) in breast tumor were detected, suggesting possible differences in HPV-induced carcinogenesis in particular organs [12]. Extreme differences in the presence of HPVs in BC samples including HPV DNA and mRNA transcripts cause that the aferomentioned “hit and run” theory is still controversial.

E6 promotes p53 degradation via its association with E6-AP, an auxiliary protein in the ubiquitin proteolytic pathway [93]. In addition, E6 interacts physically and functionally with the cellular telomerase complex [94]. E7 proteins found in high-risk HPVs bind to pRb and other pocket proteins, including p107 and p130, thereby disrupting normal cell cycle and trigger cell proliferation [95,96]. Consequently, genomic instability results in the transformation of normal cells into cancerous cells. As shown in an in vitro model, the expression of high-risk HPV oncogenes E6 and E7 leads to the immortalization of human mammary epithelial cells [97]. Disruption of normal cell cycle via E6 and E7 oncoproteins is also associated with overexpression of cyclin dependent kinase inhibitor 24 (CDKN2A or p16INK4A) often used as marker of cervical carcinogenesis. However, CDKN2A has not been shown as a good surrogate marker for HPV infection in breast cancer tissue [67]. Moreover, E6 and E7 interact with the major tumor-suppressor genes BRCA 1 and BRCA 2 [98].

Several cellular pathways are involved in the transformation of mammary epithelial cells associated with HPV E6 and E7 proteins. These proteins inhibit pRb, p53, NFX1 and BRCA1, leading to an upregulation of nuclear factor kappa B and nuclear factor kappa E pathways [99,100,101]. It has also been shown that HPV E6 and E7 proteins are capable of promoting the proliferation of BC cells, inhibit apoptosis by upregulating BCL2 and stabilizing the HER2 receptor [84,102]. The presence of HPV may also alter the expression of a member of the cytidine deaminase gene family APOBEC3B (A3B), and increase the production of reactive oxygen species [87,103,104]. According to genome-wide association studies and studies based on the The Cancer Genome Atlas (TCGA) datasets, the APOBEC-associated mutation signatures in BC were more common in East Asians (31.2%) and less common in Europeans (9.0%) and West Africans (4.2%) [105,106]. The APOBEC3A and APOBEC3B proteins may promote particular mutations in cancer genomes, a phenomenon known as APOBEC mutagenesis. Several variables, including genetic and environmental factors, impact this mutation pattern in individuals with bladder and breast cancer [107]. Furthermore, the HPV-triggered activation of STAT3 is implicated in the pro-inflammatory cytokine gene expression in breast and cervical cancer [108].

Viruses contribute to carcinogenic processes by a variety of mechanisms, including chronic inflammation, disruption of cellular regulatory mechanisms and resistance to apoptosis. Chronic inflammation is associated with an increase in cytokines such as transforming growth factor beta (TGF-1) and interleukins (IL), resulting in a proliferation of breast tumors [109]. Furthermore, HPV infection is associated with inflammatory cytokines (IL-1, IL-6, IL-17, TGF-β and TNF-α) and transcription factor NF-kB [110].

There is also the possibility that HPV could function synergistically with the estrogen receptor (ER) signaling pathway. Specifically, Wu et al. demonstrated that the E2 protein cooperates with nuclear receptor coactivators in order to enhance the ERE-dependent transcriptional activity of ERα [111]. Therefore, high estrogen signaling caused by overexpression of the ER gene may contribute to the overexpression of HPV genes E6 and E7 in HPV-positive BC cells, thereby enhancing the development and progression of the disease [8].

## 4. Research Analyzing the Association of HPV and Breast Cancer

A well-established relationship exists between HPV and cancers of the uterine cervix, anogenital region, head and neck, and skin. A variety of other cancers have been reported to be associated with HPV, including glioblastoma, colorectal, lung and breast cancers; however, its pathogenic role remains ambiguous and controversial [112,113,114,115].

No definitive link has been established yet between HPV and BC. Although the presence of HPV alone is insufficient to cause breast cancer development, it is anticipated to be an early trigger followed by cumulative alterations over time. HPV DNA-containing cells have been identified even in the tissues surrounding BC (normal tissue) [116].

Multiple studies used primers for the detection of dozens of HPV types, including lrHPV and the whole spectrum of hrHPV types (Table 1 and Table 2). HPV16 is the most common genotype detected in both benign and breast cancer tumors. Moreover, the incidence of HPV 16 variants is different between cervical and breast cancers, indicating the possible tissue-specific HPV16 variants [117]. The most common HPV types, responsible for 70% of all HPV-related BC cases globally, are HPV 16, 18 and 33 [118]; however, the presence of low-risk types (HPV6, 11, 23 and 124) has also been reported [7,40]. In American BC patients, HPV16 was also the most prevalent, whereas HPV18 and HPV33 were more common in Australian and Chinese BC patients [119]. Other HPVs often present in BC samples include types 31, 39, 45, 52 and 58 [57,70,71]. Geographic location, sample size and methodological differences may account for the difference in HPV prevalence and genotype distribution [120].

A study conducted by Band et al. in 1990 suggested that HPV infection may be associated with BC. In their study, HPV was found to immortalize normal human mammary epithelial cells and to decrease the need for growth factors in these cells [121]. By identifying HPV in 29.4% of BC samples in 1992, Di Lonardo et al. demonstrated for the first time the potential role of HPV in BC pathogenesis [122]. In addition to BC tissue, HPV infection has also been reported in adjacent normal and benign breast tissues (e.g., intraductal papilloma), nipple tissue, breast ductal lavages, nipple discharges and breast milk [11,17,26,61].

A study conducted by Haghshenas et al. revealed that 23.6% of breast cancer cases were infected with HPV [118]. According to a recent research analyzing 2211 samples of BC, the prevalence of HPV was 23%, ranging from 13.4% in Europe to 42.9% in North America and Australia [123]. Furthermore, two other meta-analyses found similar results (24.49% and 30.30% positivity for HPV DNA in BC tissues) [80,124]. In light of the results of Gupta et al., which revealed that HPV and Epstein–Barr virus (EBV) were co-present in 47 % of samples, things become more complex. Both viruses can be detected in aggressive types of BC [68]. In addition, a few cases showed the co-presence of HPV, EBV and MMTV-like virus, with possible interactions between these viruses occurring in breast carcinogenesis [125].

In the analysis of 37 case–control studies with 3607 BC cases and 1728 controls, there was conclusive evidence that BC risk was increased in cases of HPV positivity (summary odds ratio (SOR) = 6.22, 95%CI: 4.25–9.12) [126]. The calculated risks varied among the available meta-analyses: HR 1.18 (95%CI: 1.15–1.21) [127], OR 4.02 (95%CI: 2.42–6.68) [128], and OR 5.43 (95%CI: 3.24–9.12) [48].

Fewer studies showed no HPV in breast cancer tissue [76,129]. However, most published studies have indicated the presence of HPV in BC. Globally, the published research has found substantial variation in the presence of HPV in BC. Differences in HPV detection are attributed to demographic factors, sample type (e.g., paraffin-embedded tissue vs. fresh frozen tissue) and the different sensitivity levels of the methods used to detect HPV.

### 4.1. HPV DNA and Breast Cancer Types

HPV DNA is more frequently present in BC tissues compared to benign breast tumors or normal breast tissues [130]. Several studies have demonstrated that HPV DNA is more prevalent in TNBC and HER2+ BC than in luminal types of BC. Furthermore, among the luminal subtypes, HPV DNA was found in younger BC patients and in BC tissues that were significantly Ki67-positive, higher grade and had lymph node invasion [11,30,131,132]. As a result of these findings, it appears that aggressive breast tumors contain HPV DNA. While HPV DNA alone is not sufficient to initiate the carcinogenic process, it is believed that, as a component of the environment, HPV DNA may contribute to defining the BC tumorigenic phenotype [30].

### 4.2. Presence of HPV DNA in BC Tissue in Correlation with Previous Cervical Dysplasia

A number of studies have shown an increased incidence of breast cancer among patients with a history of cervical dysplasia, indicating a possible link between uterine cervix infection and breast glandular tissue infection [43]. Almost half of women with a history of HPV-16-positive high-grade cervical lesions showed correlations with HPV DNA presence in diagnosed breast cancer [33]. Based on the research of Widschwendter et al. and Damin et al., HPV-16 DNA can be detected more frequently in women with BC who have had cervical cancer in the past [133,134]. Similarly, different meta-analysis found that HPV-related BC was associated with a history of high-grade cervical cancer or CIN with a OR of 7.98 (95%CI: 1.84–34.67) [135]. Hansen et al. found that standardized incidence ratios (SIRs) of BC are higher in women with a history of squamous or glandular dysplasia than in women without such a history (squamous lesions SIR 1.10 (95%CI: 1.05–1.14) and glandular lesions SIR 1.52 (95%CI: 1.11–2.08)) [136]. Similarly, conization in the patient’s history was related to an increased risk of BC (SIR 1.10 (95%CI: 1.0–1.1)). BC risk was elevated throughout the follow-up, especially in the first five years (SIR 1.20 (95%CI: 0.92–1.5) [137].

### 4.3. Heterogeneity of the Research

Due to low viral load of HPV and a variety of diagnostic techniques, it is often difficult to determine whether HPV is present in tissue samples. These diagnostic techniques include in situ hybridization (ISH), different subtypes of polymerase chain reaction (PCR) and next-generation sequencing (NGS) [73]. Conflicting results may also be attributed to the original tissue samples (paraffin-embedded versus fresh tissue) since HPV present in long-stored paraffin-embedded tissues may be destroyed during sample processing and fixation [116]. Furthermore, the equally important fact is that PCR cannot distinguish among the types of cells that are infected. Thus, PCR may lead to an inaccurate assessment of the relationship between HPV infection and BC [138]. Additional possible sources of false positive results include amplicon contamination, antibody cross-reactivity with undesired antigens and strong background staining in detection systems. It is possible to obtain false negative results due to test insensitivity, poor antigen retrieval methods or improper tissue fixation and preparation [73]. All the possible factors affecting HPV diagnostics in breast tissue are summarized in Table 3.

## 5. Discussion

A possible role for oncogenic HPV types in breast carcinogenesis was suggested after Band et al. showed that plasmids containing HPV 16 and 18 could immortalize mammary epithelial cells [121]. The fact that mammary pathologies are almost exclusively glandular in origin, while HPV-associated malignancies are dominantly squamous, is a significant argument against the association [142].

Despite the fact that approximately 30 years have passed since this hypothesis was first proposed, its definitive conclusion remains unknown [126]. There was a significantly higher prevalence of HPV infection in BC samples when compared to adjacent normal tissue, fibroadenomas, fibrocystic changes, mastitis, intraductal papillomas and breast [8]. The studies by Doosti et al. [49], Bakhtiyrizadeh et al. [53] and Gannon et al. [79] showed that HPV DNA was not found in breast cancer tissues, whereas Cavalcante et al. [57] found high HPV prevalence rates. Baltzell et al. used in situ polymerase chain reaction (IS PCR) compared to frequently used solution PCR techniques (standard or nested). Their goal was to eliminate the possibility of specimen HPV-16 being consequently detected only in a small percentage of cases (2.9 % for PCR and 5.7 % for ISH) [38]. In light of these conflicting results, we theorize that HPV may not be a causative agent for all BC lesions but rather acts as a cofactor and modulator. In the case of cervical carcinogenesis, persistent HPV infection was shown to be the major risk factor of contracting the disease. Multiple modifiable and non-modifiable factors also affect the HPV and its oncogenic potential. Therefore, it is unlikely that HPV can cause breast cancer directly. Furthermore, to establish a link between HPV and BC, these criteria must be met [116]:

- HPV should be more prevalent in breast cancer cases than in normal samples;
- Exposure to the HPV should precede disease outcome;
- Multidisciplinary research should be conducted to replicate the association between HPV and breast cancer;
- A dose–response relationship should exist between exposure levels and the incidence of BC;
- Viral causality should be explained in terms of the mode of transmission and the natural history and pathology;
- The virus can infect and transform mammary epithelium and induce cancer in an animal model;
- The virus can induce cancer in an animal model by infecting and transforming the mammary epithelium;
- Preventing HPV infection should reduce the incidence of cervical cancer.

The results of the study of Murtaza et al. failed to establish a casual association between HPV and BC according to the Bradford Hill criteria (nine criteria to provide epidemiologic evidence of a causal relationship between a presumed cause and an observed effect). In their study, HPV was proposed as a cause–effect agent or at least as one of the co-factors involved in the pathogenesis of BC [143].

With the current state of the knowledge, how we can be sure that we are not looking at false negative or positive results? It is possible to obtain false negative results due to formalin-induced DNA fragmentation [140], DNA quality testing missing [130], inadequate virus detection assays [11], incorrect primers or hybridization probes [144] and the laser microdissection of cells [145]. Conversely, false positive results may be caused by a lack of quality in sequencing procedures as well as contamination, which is a major concern among all virologists. Cells brought into the sample by manipulation (e.g., dermal cells) could be the source of contaminated samples [146]. HPV is detectable in 18% of fomites [147] and can survive for up to seven days on environmental surfaces after desiccation [148,149]. Another factor that could suggest contaminated samples is a high prevalence of HPV in the control and benign breast samples [150]. The use of in situ hybridization methods, such as CISH and PCR-ISH, is associated with a decrease in the possibility of false positive results [116]. Chromogenic in situ hybridization allows for a topographic visualization of HPV within the nuclei of tumor cells, indicating the integration of viral DNA into host DNA, which is the first step of malignant transformation [67].

## 6. Conclusions

The possible presence of HPV in unusual body tissues, such as the female breast, remains elusive. Data regarding the presence of HPV DNA in tumor samples from patients with BC are very inconsistent, and there is no clear indication of how HPV is transmitted to the breast. However, the presence of HPV in BC cannot be denied. An interesting fact is that HPV DNA can be found in healthy and benign breast tissue, which may prove useful in observing if those chosen patients develop BC in the future. These findings lend support to the hypothesis that the HPV contributes to the development of breast cancer. Similar to the above hypothesis, the use of the HPV vaccine may contribute to a reduction in BC cases. To date, no study has attempted to correlate HPV vaccine use with BC incidence, as such studies would require a much longer period of time to be conducted. For a better understanding of the role of the HPV in BC, extensive studies with a larger sample size and unified diagnostic methods are required, considering the complexity involved and the relatively low prevalence of HPV infection in BC lesions.

## Figures and Tables

**Table 1:** Studies focused on the association of BC and HPV published since 2010 (FFPE tissue samples).

Study	Method	No. of Histological Samples	HPV Positivity	HPV Types Detected
Aguayo et al., 2011 [34]	PCR, Inno-Lipa-HPV 16	BC: 55	8.7%	16
Frega et al., 2011 [35]	PCR, INNO-LiPA	BC: 31 Benign lesions: 12	29% 0%	16, 6
Herrera-Goepfert et al., 2011 [36]	PCR, INNO-LiPA	BC: 69	24%	16
Silva et al., 2011 [37]	PCR–HPV 6, 11, 16, 18	BC: 79	No positive results	n.a.
Baltzell et al., 2012 [38]	IS-PCR, IHC–12 hrHPVs	BC: 70	2.9 % (IS-PCR) 5.7 % (ISH)	16
Herrera-Romano et al., 2012 [39]	PCR	BC: 118 nipple lesions: 2	No positive results	n.a.
Sigaroodi et al., 2012 [40]	PCR	BC: 79 Benign lesions: 51	25.9% 2.4%	16, 18, 23, 6, 11, 15, 124
Eslamifar et al., 2015 [41]	PCR	BC: 100 Healthy controls: 50	No positive results	n.a.
Fu et al., 2015 [42]	PCR and ISH–HPV 58, HPV 58 E7 DNA	BC: 169 Benign lesions: 83	PCR/ISH–14.79%/10.06% PCR/ISH–1.2%/1.2%	58
Lawson et al., 2015 [43]	IS-PCR, NGS	BC: 855	3.5% (lrHPV) 2.3% (hrHPV)	18, 16, 52, 113
Li et al., 2015 [44]	PCR–HPV 16, 18 HPV 16 E6, E7 HPV 18 E6, E7	BC: 187 Adjacent tissue: 187 Benign lesions: 92	1.6% 0% 0%	16, 18
Ngan et al., 2015 [45]	HPV E7 IHC, PCR	sets of benign and subsequent BC specimens: 32 healthy controls: 20	72% (benign specimens) 62.5% (subsequent BC) 10%	16, 18, 45, 58
Vernet-Tomas 2015 [46]	PCR, DEIA 54 mucosal HPV types	BC: 76 Benign lesions: 2	No positive results	n.a.
Chen et al., 2016 [47]	PCR-HPV 16, 18 oncogens E6, E7	BC: 76	23.68% for HPV18 E7 6.58% for HPV18 E6 all samples negative for HPV16 E6/E7	18
Choi et al., 2016 [48]	PCR-28 hrHPVs and lrHPVs	BC: 123 Intraductal papillomas: 9 Nipple tissues: 13	17.9% 22.2% 0 %	51, 53, 40
Doosti et al., 2016 [49]	PCR	BC: 87 benign lesions: 84	22.9% 0%	16, 18, 6, 11
Ilahi et al., 2016 [50]	PCR–HPV 16 and 18	BC: 46	17.3%	16
Karimi et al., 2016 [51]	PCR–HPV 16, 18, 31, 33	BC: 70 benign lesions: 70	2.6% 0%	18
Wang et al.,2016 [52]	ISH for HPV DNA and mRNA (HPV 16,18,58)	BC: 146 Benign lesions: 83	35.6% 3.6%	16, 18, 58
Bakhtiyrizadeh et al., 2017 [53]	PCR	BC: 150 Benign lesions: 150	No positive results	n.a.
Delgado- Garcìa et al., 2017 [7]	PCR	BC: 251 Benign lesions: 186	51.8% 26.3%	16, 51, 89 as the most prevalent
Naushad et al., 2017 [54]	PCR	BC: 250 Benign tissue: 15	18,1% n.a.	n.a.
Rezaei et al., 2017 [55]	PCR, ARMS-PCR -HPV 16, 18, 31, 11, 33, 35	BC (familial): 38 BC (non-familial): 46	44.73% 26.08%	16, 18, 11 as the most prevalent
Bønløkke et al., 2018 [56]	SPF_10_ PCR-DEIA-LiPA_25_ assay–25 hrHPV and lrHPV	BC with prior dysplasia: 93 BC without prior dysplasia: 100	2.1% 1.0%	16, 56
Cavalcante et al., 2018 [57]	PCR–11 HPV types	BC: 103 Healthy tissue: 90	49.5% 15.8%	6, 11, 18, 31, 33, 52
De Carolis et al., 2018 [26]	PCR–16 HPV types	Intraductal papilloma: 10 DCIS: 9 BC: 10	40% 11.1% 30%	16, 18, 33, 51, 53
Ghaffari et al., 2018 [58]	PCR, microarray–35 hrHPV and lrHPV types	BC:72	5.52%	n.a.
Habyarimana et al., 2018 [59]	PCR	BC: 47	46.8%	16, 33, 31 as the most prevalent
Malekpour Afshar et al., 2018 [60]	PCR, INNO-LiPA	BC: 98 Benign lesions: 40	8.2% No positive results	16, 18 as the most prevalent
Balci et al., 2019 [61]	PCR	BC: 18 Breast papillomas: 27	44.4% 29.6%	11, 39 as the most prevalent
De Carolis et al., 2019 [30]	CISH (HPV 16,18), PCR (16 HPV types), NGS	BC: 273	30.4%	16, 18 as the most prevalent
Biesaga et al., 2021 [62]	PCR-21 HPV types	BC: 383	4.4%	16
Boumba et al., 2021 [63]	PCR–14 hrHPV types	BC:40	15%	16 as the most prevalent
Elagali et al., 2021 [64]	PCR	BC: 150	8.7%	16, 58, 18, 11
Gebregzabher et al., 2021 [65]	PCR–19 hrHPVs, 9 lr HPVs	BC: 75	2.7%	16, 6
Golrokh Mofrad et al., 2021 [66]	PCR	BC: 59 Benign lesions: 11	11.8% No positive results	18, 6
Guo et al., 2021 [67]	CISH–HPV 6, 11, 18, 18	BC: 90 Intraductal papillomas: 33 Healthy tissue: 33	21.1% (HPV 6,11), 43.3% (HPV 16, 18) 3.0% (HPV 6,11), 18.8% (HPV 16, 18) 0% (HPV 6, 11), 9.1% (HPV 16, 18)	16, 18, 6, 11
Gupta et al., 2021 [68]	PCR–14 hrHPV types	TNBC: 70 Healthy tissues: 14	53% 37.5%	52, 45, 31, 58, 68
Metwally et al., 2021 [69]	PCR	BC:40	17.5%	n.a.
Nagi et al., 2021 [70]	PCR–14 hrHPV types, TMA	BC: 102 Healthy tissue: 14	65% 35.6%	52, 35, 58, 45, 16 and 51 as the most prevalent
Alinezhadi et al., 2022 [71]	PCR	BC: 63 Benign lesions: 32	17.89% 28.12%	11, 16, 31, 33
De Oliveira et al., 2022 [32]	PCR	BC: 75	0%	n.a.
Gupta et al., 2022 [72]	PCR–11 hrHPV types	BC:74	65%	n.a.
Maldonado-Rodriguèz et al., 2022 [73]	PCR–32 hrHPV and lrHPV types	BC: 59 Benign lesions: 46 Healthy tissue: 11	20.3% 34.8% 27.3%	42, 31, 59 as the most prevalent

HPV, Human Papillomavirus; hrHPV, high-risk Human Papillomavirus; lrHPV, low-risk Human Papillomavirus; BC, Breast Cancer; TNBC, Triple Negative Breast Cancer; DCIS, Ductal Carcinoma In Situ; PCR, Polymerase Chain Reaction; IHC, Immunohistochemistry; FFPE, Formalin Fixed Paraffin Embedded; CISH, Chromogenic In Situ Hybdridization; NGS, Next Generation Sequencing; ARMS–Amplification-Refractory Mutation System; DEIA, DNA Enzyme Immunoassay; IS-PCR, In Situ Polymerase Chain Reaction; mRNA, mediator Ribonucleic Acid; n.a., not applicable/not available.

**Table 2:** Studies focused on the association of BC and HPV published since 2010 (fresh-frozen tissue samples).

Study	Method	No. of Histological Samples	HPV Positivity	HPV Types Detected
Hachana et al., 2010 [74]	PCR, ISH–HPV 16, 18, 31, 33, 6, 11	BC: 123	No positive results	n.a.
Antonsson et al., 2011 [75]	PCR–16 hrHPV types	BC: 54 Healthy controls: 4	50% 25%	18
Hedau et al., 2011 [76]	PCR-HPV 16,18	BC: 228	No positive results	n.a.
Mou et al., 2011 [77]	PCR–21 hrHPV and lrHPV types	BC: 62 Benign lesions: 46	6,5% No positive results	16, 18
Herrera- Romano et al., 2012 [39]	PCR	BC: 10	No positive results	n.a.
Fernandes et al., 2015 [78]	PCR, INNO-LiPA-28 HPV types	BC: 24	41.7%	51, 33, 18, 6, 11
Gannon et al. 2015 [79]	PCR, NGS	BC: 80 Benign lesions:10	16% 10%	16, 18
Zhou et al., 2015 [80]	PCR	BC/DCIS: 77 Adjacent tissue: 77	No positive results	n.a.
Islam et al., 2017 [81]	PCR–HPV 16, 18, 33	BC (prior NACT): 272 BC (after NACT): 41 Adjacent normal tissues: 21 Benign lesions: 17	63.9% 71.0% 9.5% 47.1%	16, 18, 33
Ngamkham et al., 2017 [82]	PCR–14 hrHPV and 22 lrHPV types	BC: 350 Benign lesions: 350	4.3% 2.9%	16, 33, 18, 35, 52
Salman et al., 2017 [83]	PCR-12 hrHPV types	BC:74 Benign lesions:36	47% 31%	39, 18, 45 as the most prevalent
Wang et al., 2017 [84]	HC2–13 hrHPV types	BC:81	17.3%	n.a.
ElAmrani et al., 2018 [85]	PCR–62 lrHPV and hrHPV types	BC: 76 Benign lesions: 12	25% 8.3%	51, 52, 58, 59, 66 as the most prevalent
Kouloura et al., 2018 [86]	Microarray	BC: 201 Adjacent healthy tissue: 201	No positive results	n.a.
Khodabandehlou et al., 2019 [87]	PCR	BC: 72 Healthy tissue: 31	48.6% 16.1%	16, 18, 33, 6, 11
Sher et al., 2020 [88]	PCR–12 hrHPV types	BC:50 Benign lesions: 100	10% 8%	16, 35, 58
Charostad et al., 2021 [89]	PCR	BC:36 Adjacent healthy tissue: 36	33.3% 5.5%	16, 18, 31, 6
El-Sheikh et al., 2021 [90]	PCR–HPV 16, 18, 31	BC: 72 Benign lesions: 15	22.2% No positive results	16, 18
Metwally et al., 2021 [69]	PCR	BC (fresh tissue): 40	50%	n.a.
Calderon et al., 2022 [91]	PCR	BC: 447 Benign lesions: 79	2.9% 1.3%	16, 18

HPV, Human Papillomavirus; hrHPV, high-risk Human Papillomavirus; lrHPV, low-risk Human Papillomavirus; BC, Breast Cancer; DCIS, Ductal Carcinoma In Situ; PCR, Polymerase Chain Reaction; IHC, Immunohistochemistry; NGS, Next Generation Sequencing; n.a., not applicable/not available; NACT, neoadjuvant chemotherapy.

**Table 3:** Factors affecting the HPV diagnostics in breast tissue.

Factors	Commentary
**Sampling**	
Sample size	Low sample size influences the statistical power of the research [139]
Type of analyzed samples (FFPE tissue, fresh-frozen tissue)	Formalin-induced DNA fragmentation in FFPE samples [140]
Age of samples (especially in case of FFPE)	Significant degradation of DNA in 4–6 years of storage [141]
Contamination	Manipulation with the sample
**Diagnostics**	
Diagnostic techniques (ISH, PCR, NGS)	It is impossible to confirm that the positive reactions are directly from mammary cells in case of PCR method [116]
Designed PCR primers	Variable sensitivity and specificity [8]
**Viral factors**	
Viral load	Extremely low viral load causes false test negativity [73]
Less common types of HPV undetected by PCR, etc.	Detection methods are in most cases used for the common hrHPV types

FFPE, formalin-fixed paraffin-embedded; ISH, in situ hybridization; PCR, polymerase chain reaction; NGS, next-generation sequencing; HPV, human papillomavirus.

## Data Availability

Not applicable.
